# Synthesis, Biological Evaluation and Structure-Activity Relationships of New Quinoxaline Derivatives as Anti-*Plasmodium falciparum* Agents

**DOI:** 10.3390/molecules19022166

**Published:** 2014-02-18

**Authors:** Ana Gil, Adriana Pabón, Silvia Galiano, Asunción Burguete, Silvia Pérez-Silanes, Eric Deharo, Antonio Monge, Ignacio Aldana

**Affiliations:** 1 Unidad de Investigación y Desarrollo de Medicamentos, Centro de Investigación en Farmacobiología Aplicada (CIFA), Universidad de Navarra, c/ Irunlarrea 1, Pamplona 31008, Spain; E-Mails: sgaliano@unav.es (S.G.); abpnqb@cid.csic.es (A.B.); sperez@unav.es (S.P.-S.); amonge@unav.es (A.M.); ialdana@unav.es (I.A.); 2 Grupo Malaria, Facultad de Medicina, Universidad de Antioquia, Medellín 050010, Colombia; E-Mail: apabon72@gmail.com; 3 Programa de Biología, Facultad de Ciencias Básicas, Universidad del Atlántico, Barranquilla 080001, Colombia; 4 Instituto de Química Avanzada de Cataluña, Consejo Superior de Investigaciones Científicas (CSIC), c/ Jordi Girona 18-26, Barcelona 08034, Spain; 5 PHARMA-DEV, UMR 152 IRD-UPS, Faculté des Sciences Pharmaceutiques, Université Paul Sabatier, 35 chemin des Maraîchers, 31062 Toulouse Cedex 09, France; E-Mail: ericdeharo@gmail.com

**Keywords:** *Plasmodium falciparum*, antimalarial agents, quinoxaline, quinoxaline 1,4-di-*N*-oxide, chalcone

## Abstract

We report the synthesis and antimalarial activities of eighteen quinoxaline and quinoxaline 1,4-di-*N*-oxide derivatives, eight of which are completely novel. Compounds **1a** and **2a** were the most active against *Plasmodium falciparum* strains. Structure-activity relationships demonstrated the importance of an enone moiety linked to the quinoxaline ring.

## 1. Introduction

Malaria is an infectious disease with an estimated 219 million cases and 660,000 deaths in 2010. Of these cases 86% correspond to children under 5 years old. In 2012, there were a total of 104 countries where malaria is considered to be endemic [1].

*Plasmodium falciparum* is the most dangerous form of the malaria parasite and it is responsible for a very high percentage of clinical attacks [2]. Artemisinin-based combination therapies (ACTs) are the standard treatment against uncomplicated *P. falciparum* malaria. Nevertheless, resistance to ACTs containing dihydroartemisinin has been reported in Pailin (Cambodia) [3] and resistance to pyrethroids (used as insecticides) has been detected in 64 countries around the World [1]. Other antimalarial drugs, such as chloroquine or mefloquine, are not effective enough [4]. FCR-3 *P. falciparum* is a chloroquine-resistant strain, but sensitive to pyrimethamine and sulfadoxine [5].

Over the last years, our group has been working on the development and synthesis of new quinoxaline derivatives. As a result of this line of research, the activities of quinoxaline 1,4-di-*N*-oxide derivatives against *Mycobacterium tuberculosis* [6,7,8,9,10,11,12,13], *Trypanosoma cruzi* [14,15], *Leishmania amazonensis* [16], *L. infantum* [17], *P. falciparum* [16,17,18,19,20,21,22,23] and different tumor cells [24,25,26] have been reported. The presence of two *N*-oxides is associated with a significant increase in some biological properties, such as anticancer [27] or antioxidant [28] activity. On the other hand, some reduced quinoxalines showed good antitubercular activity [29].

**Figure 1:**
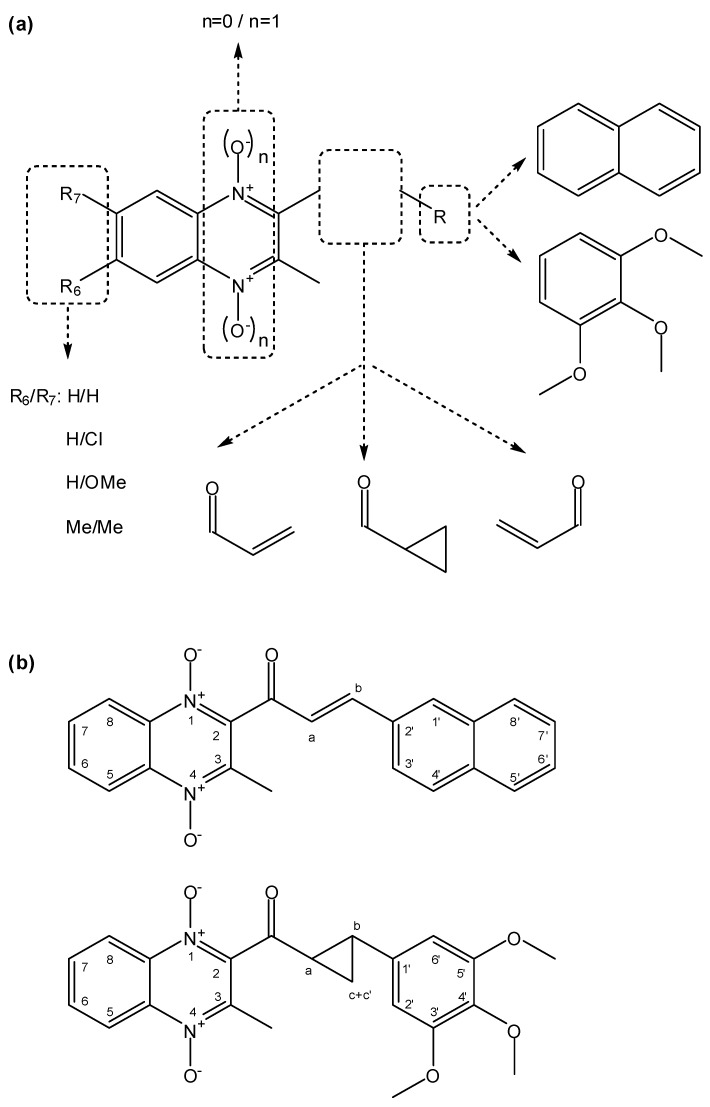
(**a**) Design of quinoxaline derivatives as potential antimalarial drugs. (**b**) Examples of two numbered compounds obtained in this synthesis.

In this article, we report the synthesis and antimalarial activity of some quinoxaline analogs of chalcones and other compounds derived from them (Figure 1). Chalcones or 1,3-diaryl-2-propen-1-ones are α,β-unsaturated ketones with a large number of biological activities [30]. Different chalcones have been reported as anti-inflammatory [31], anticancer [32], antitubercular [33,34] or antimalarial [34,35,36,37,38] agents. With regard to antiplasmodial activity, previous structure-activity relationship studies found that the enone linker [38] and its *trans*-configuration [39] are essential in active chalcones.

Some of the compounds belonging to this study were previously tested against *L. amazonensis* [40] (series 1), *T. cruzi* and *L. peruviana* [41] (series 3) and as cytotoxic agents [42] (compound **1a**) and anti-inflammatory/antioxidant [28,43] agents (series 1, 3 and 7). According to the structure-activity relationships, these reports affirm that compounds in series 3 have better antioxidant activity than their analogs in series 1 due to the fact that the former group of molecules lacks the *N*-oxide groups [28]. On the other hand, compounds **1d** and **3d** have an interesting activity against different *Leishmania* strains, so the activity is associated with R_6_/R_7_ = Me/Me substitution [40,41]. Compound **1a** stands out as both a good anti-inflammatory and cytotoxic agent [42,43].

With the aim of expanding the SAR study of these chalcone analogs and obtaining new compounds with improved antimalarial activity, we describe herein the synthesis and the relationships between structure and antiplasmodial activity against the FCR-3 *P. falciparum* strain of eighteen quinoxaline and quinoxaline 1,4-di-*N*-oxide derivatives.

## 2. Results and Discussion

### 2.1. Chemistry

Synthesis of compounds in series 1, 3 and 7 were previously reported [25,40]. Benzofuroxans **I** with R_a_/R_b_ = H/H, H/Cl and H/OMe are commercially available. Benzofuroxan with R_a_/R_b_ = Me/Me was synthesized by an oxidative cyclization of 4,5-dimethyl-2-nitroaniline according to a previously described method [44].

**Scheme 1:**
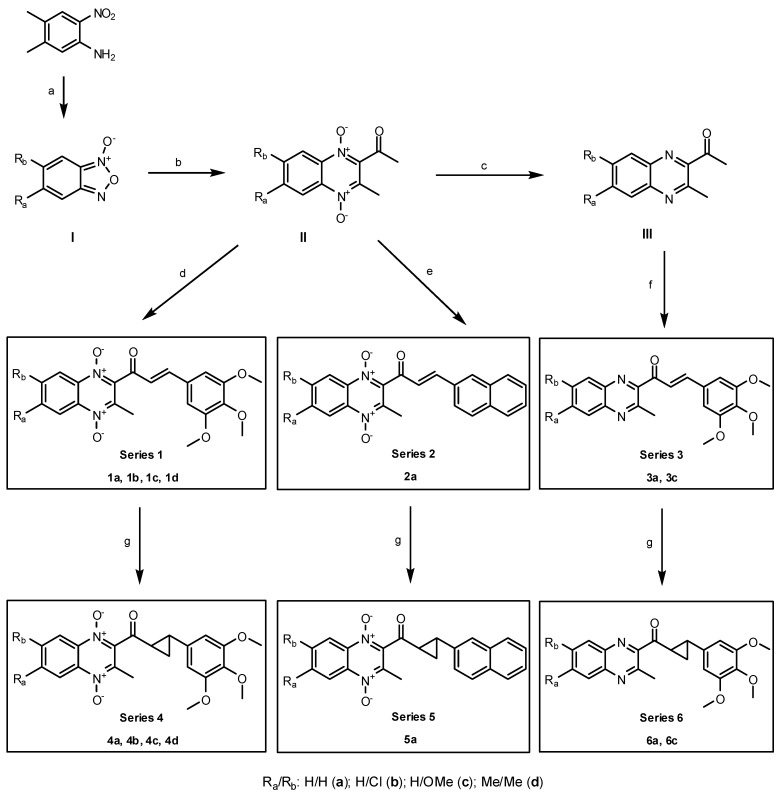
Synthesis of series 1, 2, 3, 4, 5 and 6.

Scheme 1 shows the entire synthetic route for obtaining compounds **1a**–**d**, **2a** and **3a**, **3c** and their cyclopropyl derivatives **4a**–**d**, **5a** and **6a**, **6c**. The starting reagents used, quinoxaline 1,4-di-*N*-oxides **II** and reduced quinoxalines **III**, were obtained following previously reported procedures [7,27]. Quinoxaline 1,4-di-*N*-oxides **II** were synthesized by a classic Beirut reaction [45], using CaCl_2_ and 1-aminoethanol as the catalysts [46]. Their reduced analogs **III** were prepared by reduction of the *N*-oxide groups [27], for which a reaction temperature of 65 °C and a mixture of ethyl acetate/methanol 1:1 as the solvent [47] was established.

*Reagents and conditions*: (**a**) NaClO, *N*,*N*-DMF, 0 °C; (**b**) 2,4-pentanedione, CaCl_2_, 1-aminoethanol; (**c**) Na_2_S_2_O_4_, AcOEt/MeOH 1:1, 65 °C; (**d**) 3,4,5-trimethoxybenzaldehyde, 3% NaOH/MeOH, −10 °C; (**e**) 2-naphthaldehyde, 5% NaOH/MeOH; (**f**) 3,4,5-trimethoxybenzaldehyde, 3% NaOH/MeOH, r.t.; (**g**) TBAB, TMSOI, 15% aq. NaOH.

As previously described [48], in reactions of monosubstituted benzofuroxans 7-substituted quinoxaline 1,4-di-*N*-oxide derivatives prevail over the 6-isomers. While compound **II** with R_6_/R_7_= H/Cl was obtained as the major isomer and was isolated with a subsequent purification [25], 2-acetyl-7-methoxy-3-methylquinoxaline 1,4-di-*N*-oxide was obtained as the sole isomer [7].

Chalcone analogs **1a**–**d** were synthesized by a previously reported base-catalyzed Claisen-Schmidt condensation [49], using 3% NaOH in methanol as the catalyst and establishing an optimum reaction temperature of −10 °C [43]. Their reduced analogs **3a**, **3c** were synthesized using the same catalyst, but in this case, the condensation was performed at room temperature, as previously reported [28]. The compounds **1a**–**d** and **3a**, **3c** obtained have been previously described [28,47]. Compound **2a** was first synthesized according to a previously reported method [50]. It was a spontaneous reaction that gives the desired compound in good yield.

New cyclopropyl derivatives **4a**–**d**, **5a** and **6a**, **6c** were obtained according to a previously reported method [51]. These reactions were carried out between compounds **1a**–**d**, **2a** and **3a**, **3c** and trimethylsulfoxonium iodide (TMSOI) in the presence of an aqueous solution of NaOH, with tetrabutylammonium bromide (TBAB) as a phase transfer catalyst and dichloromethane as the solvent. With regard to the reaction mechanism, Corey and Chaykovsky [52] demonstrated that trimethylsulfoxonium halides in the presence of a strong base allow the formation of a reactive compound named dimethylsulfoxonium methylide (DMSY), commonly known as Corey’s reagent [53]. The reaction of DMSY with an α,β-unsaturated ketone involves the cyclopropanation of C=C double bond [54].

As their coupling constant values in the ^1^H-NMR spectra showed *J* values ≈ 16 Hz [47], the double bond geometries of compounds **1a**–**d** and **3a**, **3c** were *trans*, just like the newly synthesized **2a**. According to previous reports [55,56], the Corey-Chaykovsky reaction with TMSOI ocurrs with retention of stereochemistry, giving *trans*-cyclopropyl derivatives **4a**–**d**, **5a** and **6a**, **6c** [51].

**Scheme 2:**
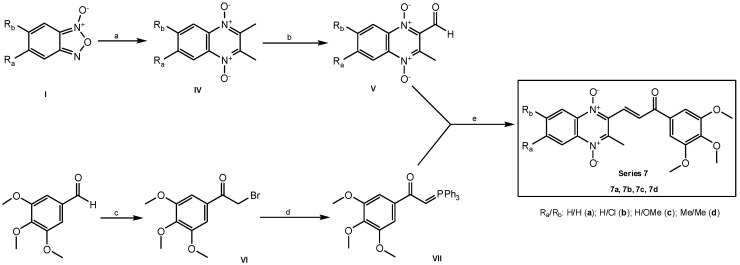
Synthesis of series 7 [28].

Scheme 2 shows the synthetic route for obtaining compounds **7a**–**d**. These chalcone analogs were obtained by a microwave-assisted Wittig reaction [28] between 2-formyl-3-methylquinoxaline 1,4-di-*N*-oxide derivatives **V** and a phosphonium ylide **VII**, both previously synthesized [28,45,57].

*Reagents and conditions*: (**a**) 2-butanone, morpholine; (**b**) SeO_2_, acetonitrile, MW: 5 min, 200 W; (**c**) Br_2_, acetic acid; (**d**) PPh_3_, NaH/H_2_O, toluene; (**e**) MeOH, MW: 5 min, 25 W.

### 2.2. Biological Results

In this study, eighteen quinoxaline and quinoxaline 1,4-di-*N*-oxide derivatives were tested against a chloroquine-resistant FCR-3 strain of *P. falciparum*. All the synthesized compounds and their biological activities are shown in Table 1, Table 2 and Table 3.

**Table 1:** *In vitro* activities of chalcone derivatives. 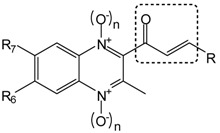

Compound	R_6_	R_7_	n	R	IC_50_ (μM) ^a^	SD ^b^
**1a**	H	H	1	3,4,5-trimethoxyphenyl	6.2	1.7
**1b**	H	Cl	1	3,4,5-trimethoxyphenyl	27.3	3.6
**1c**	H	OMe	1	3,4,5-trimethoxyphenyl	31.1	20.4
**1d**	Me	Me	1	3,4,5-trimethoxyphenyl	24.4	14.0
**2a**	H	H	1	naphthyl	5.8	0.8
**3a**	H	H	0	3,4,5-trimethoxyphenyl	21.5	5.3
**3c**	H	OMe	0	3,4,5-trimethoxyphenyl	26.4	0.3
CQ ^c^	-	-	-	-	0.173	0.003

^a^ IC_50_, concentration required for inhibiting growth of FCR-3 *P. falciparum* strain by 50% (in μM). Mean values of two independent experiments performed in triplicate; ^b^ SD, standard deviation; ^c^ Chloroquine, control.

**Table 2:** *In vitro* activities of cyclopropyl derivatives. 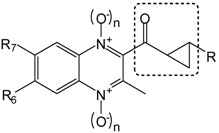

Compound	R_6_	R_7_	n	R	IC_50_ (μM) ^a^	SD ^b^
**4a**	H	H	1	3,4,5-trimethoxyphenyl	56.4	6.4
**4b**	H	Cl	1	3,4,5-trimethoxyphenyl	20.1	6.9
**4c**	H	OMe	1	3,4,5-trimethoxyphenyl	NA ^d^	-
**4d**	Me	Me	1	3,4,5-trimethoxyphenyl	NA	-
**5a**	H	H	1	naphthyl	62.1	20.0
**6a**	H	H	0	3,4,5-trimethoxyphenyl	NA	-
**6c**	H	OMe	0	3,4,5-trimethoxyphenyl	NA	-
CQ ^c^	-	-	-	-	0.173	0.003

^a^ IC_50_, concentration required for inhibiting growth of FCR-3 *P. falciparum* strain by 50% (in μM). Mean values of two independent experiments performed in triplicate; ^b^ SD, standard deviation; ^c^ Chloroquine, control; ^d^ NA, not active.

**Table 3:** *In vitro* activities of inverted chalcone derivatives. 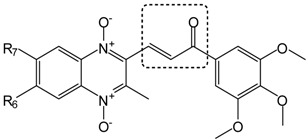

Compound	R_6_	R_7_	IC_50_ (μM) ^a^	SD ^b^
**7a**	H	H	29.0	3.5
**7b**	H	Cl	24.2	0.8
**7c**	H	OMe	34.3	2.0
**7d**	Me	Me	24.5	4.7
CQ ^c^	-	-	0.173	0.003

^a^ IC_50_, concentration required for inhibiting growth of FCR-3 *P. falciparum* strain by 50% (in μM). Mean values of two independent experiments performed in triplicate; ^b^ SD, standard deviation; ^c^ Chloroquine, control.

Almost all the synthesized compounds exhibited some activity against *P. falciparum*, but none of them had better IC_50_ values than chloroquine itself. Compounds **1a** and **2a** have been chosen as lead compounds with interesting activities against the parasite. Although both leads were chalcone analogs with R_6_/R_7_ = H/H, compounds **7b** and **4b** had the best activities among inverted chalcone and cyclopropyl derivatives. In these cases, the presence of a halogen atom in position 7 led to an increase in the activity. In fact, **4b** was the third most active compound of the study. These results coincide with previous reports [18], where the most active compounds were those without any substitution on the quinoxaline ring and with monosubstitution by an electron withdrawing group.

Apart from compound **4b**, changing the double bond in chalcones (compounds **1a**–**d**, **2a** and **3a**, **3c**) for a cyclopropyl structure led to a dramatic drop in the antiplasmodial activity in each case (compounds **4a**–**d**, **5a** and **6a, 6c**). The same trend was noted when comparing cyclopropyl derivatives **4a**–**d** with their analogs in series 7.

With regard to series 1 and 7, the inversion of the chalcone did not lead to a change in the antimalarial activity, except for compound **1a**, whose IC_50_ was significantly lower than that of the others. Therefore, the orientation of the enone moiety does not appear to be a determining factor for the antimalarial activity. Finally, only reduced chalcones **3a** and **3c** showed some interesting activity, while reduced cyclopropyl derivatives **6a** and **6c** were completely inactive. According to previous reports [58], a minimum requirement for the antimalarial activity was the oxygenation of *N*-1 and *N*-4 of the quinoxaline ring, because mono and reduced compounds were not active. Nevertheless, in this study the presence of *N*-oxides was not decisive for the synthesized compounds to be active.

## 3. Experimental

### 3.1. General Information

All of the synthesized compounds were chemically characterized by thin layer chromatography (TLC), melting point, proton nuclear magnetic resonance (^1^H-NMR), infrared spectroscopy (IR) and elemental microanalyses (CHN). Alugram SIL G/UV254 (0.2 mm layer, Macherey-Nagel GmbH & Co. KG., Düren, Germany) was used for TLC, and silica gel 60 (0.040–0.063 mm, Merck, Darmstadt, Germany) was used for column chromatography. The ^1^H-NMR spectra were recorded on a Bruker 400 Ultrashield instrument (400 MHz, Bruker, Billerica, MA, USA), using TMS as the internal standard and DMSO-*d_6_* as the solvent. Chemical shifts are reported in ppm (*δ*) and coupling constants (*J*) values are given in Hertz (Hz). Signal multiplicities are represented by: s (singlet), d (doublet), t (triplet), q (quadruplet), dd (double doublet) and m (multiplet). The IR spectra were recorded on a Thermo Nicolet Nexus FTIR (Madison, WI, USA) in KBr pellets. Frequencies (*ν*) are reported in cm^−1^ and peak intensities are represented by: w (weak), m (medium), s (strong) and vs (very strong). Elemental microanalyses were obtained on a CHN-900 Elemental Analyzer (Leco, Tres Cantos, Madrid, Spain) from vacuum-dried samples. The analytical results for C, H and N were within ±0.4 of the theoretical values, indicating a purity of >95%. All reagents and solvents were purchased from commercial sources: E. Merck (Darmstadt, Germany), Panreac Química S.A. (Barcelona, Spain), Sigma-Aldrich Química S.A. (Alcobendas, Madrid, Spain), Acros Organics (Janssen Pharmaceuticalaan, Geel, Belgium), Scharlau (F.E.R.O.S.A., Barcelona, Spain) and Lancaster (Bischheim-Strasbourg, France).

### 3.2. General Procedure of Synthesis

#### 3.2.1. Synthesis of 3-Methyl-2-[3-(naphth-2-yl-prop-2-enoyl)] quinoxaline 1,4-di-*N*-oxide Derivatives (Series 2)

The appropriate quinoxaline di-*N*-oxide **II** (2-acetyl-3-methylquinoxaline 1,4-di-*N*-oxide, 3 mmol) and 2-naphthaldehyde (3 mmol) were dissolved in methanol (30 mL). 5% NaOH in methanol (10 mL) was added dropwise and the reaction mixture was magnetically stirred at room temperature for 15 min, until a yellow precipitate appeared. The solid obtained was filtered off and washed with diethyl ether.

*3-Methyl-2-[3-(naphth-2-yl-prop-2-enoyl)] quinoxaline 1,4-di-*N*-oxide* (**2a**). Yield 57%. mp 184 °C. ^1^H-NMR (DMSO-*d_6_*) δ ppm: 2.40 (s, 3H, CH_3_); 7.37 (d, 1H, H_b_, *J*_b-a,*trans*_ = 16.4 Hz); 7.58 (m, 2H, H_6_+H_7_); 7.91 (d, 1H, H_3'_); 8.01 (m, 5H, H_4'_+H_5'_+H_6'_+H_7'_+H_8'_); 8.03 (d, 1H, H_a_, *J*_a-b,*trans*_ = 16.0 Hz); 8.24 (s, 1H, H_1'_); 8.47 (d, 1H, H_8_, *J*_8–7_ = 8.6 Hz); 8.58 (d, 1H, H_5_, J_5–6_ = 8.4 Hz). IR (KBr) ν cm^−1^: 3091 (w, ν_arC-H_); 3055 (w, ν_alkC-H_); 3018 (w, ν_C-H_); 1675 (m, ν_C=O_); 1600 (s, ν_arC=C_); 1331 (vs, ν_N-O_). Anal. Calc. for C_22_H_16_N_2_O_3_: C = 74.14%, H = 4.53%; N = 7.86%; Found: C = 74.19%, H = 4.40%; N = 7.85%.

#### 3.2.2. Synthesis of 3-Methyl-2-[2-(3,4,5-trimethoxyphenyl)cyclopropanecarbonyl] quinoxaline 1,4-di-*N*-oxide Derivatives (Series 4), 3-Methyl-2-[2-(2-naphthyl)cyclopropanecarbonyl] quinoxaline 1,4-di-*N*-oxide Derivatives (Series 5) and 3-Methyl-2-[2-(3,4,5-trimethoxyphenyl)cyclopropanecarbonyl] quinoxaline Derivatives (Series 6)

The appropriate chalcone **1a**–**d**, **2a** and **3a**, **3c** (1 mmol), trimethylsulfoxonium iodide (TMSOI, 2 mmol) and tetrabutylammonium bromide (TBAB, 0.2 mmol) were dissolved in dichloromethane (40 mL) and stirred magnetically at room temperature for 15 min. Then a 15% aqueous solution of NaOH (10 mL) was added dropwise and the reaction mixture was stirred magnetically at room temperature for 24 h, until the solution turned dark. At this point, excess dichloromethane and water were added to the reaction. The organic phase was extracted, dried with anhydrous Na_2_SO_4_, filtered and concentrated under reduced pressure. The residue was purified by column chromatography using ethyl acetate as the eluent. The desired compounds **4a**–**d**, **5a** and **6a**, **6c** were obtained after recrystallization from ethanol. 

*3-Methyl-2-[2-(3,4,5-trimethoxyphenyl)cyclopropanecarbonyl] quinoxaline 1,4-di-*N*-oxide* (**4a**). Yield 56%. mp 178–179 °C. ^1^H-NMR (DMSO-*d_6_*) δ ppm: 1.86 (m, 2H, H_c_+H_c'_); 2.40 (s, 3H, CH_3_); 2.78 (m, 1H, H_a_); 2.91 (m, 1H, H_b_); 3.59 (s, 3H, *p*-OCH_3_); 3.75 (s, 6H, *m*-OCH_3_); 6.55 (s, 2H, H_2'_+H_6'_); 7.98 (m, 2H, H_6_+H_7_); 8.44 (d, 1H, H_8_, *J*_8–7_ = 8.2 Hz); 8.49 (d, 1H, H_5_, *J*_5–6_ = 8.2 Hz). IR (KBr) ν cm^−1^: 3071 (w, ν_arC-H_); 2998 and 2965 (w, ν_C-H_); 1690 (m, ν_C=O_); 1590 (m, ν_arC=C_); 1329 (s, ν_N-O_); 1128 (s, ν_C-O-C_). Anal. Calc. for C_22_H_22_N_2_O_6_: C = 64.37%, H = 5.41%; N = 6.83%; Found: C = 64.08%, H = 5.74%; N = 6.69%.

*7-Chloro-3-methyl-2-[2-(3,4,5-trimethoxyphenyl)cyclopropanecarbonyl] quinoxaline 1,4-di-*N*-oxide* (**4b**). Yield 9%. mp 143–144 °C. ^1^H-NMR (DMSO-*d_6_*) δ ppm: 1.88 (m, 2H, H_c_+H_c'_); 2.39 (s, 3H, CH_3_); 2.79 (m, 1H, H_a_); 2.87 (m, 1H, H_b_); 3.60 (s, 3H, *p*-OCH_3_); 3.76 (s, 6H, *m*-OCH_3_); 6.54 (s, 2H, H_2'_+H_6'_); 8.01, 8.02 (dd, 1H, H_6_, *J*_6–8_ = 2.3 Hz, *J*_6–5_ = 9.2 Hz); 8.42 (d, 1H, H_8_, *J*_8–6_ = 2.2 Hz); 8.48 (d, 1H, H_5_, *J*_5–6_ = 9.2 Hz). IR (KBr) ν cm^−1^: 3101 (w, ν_arC-H_); 2935 (w, ν_C-H_); 1695 (m, ν_C=O_); 1592 (m, ν_arC=C_); 1326 (s, ν_N-O_); 1128 (s, ν_C-O-C_). Anal. Calc. for C_22_H_21_N_2_O_6_Cl: C = 59.39%, H = 4.77%; N = 6.30%; Found: C = 59.52%, H = 4.93%; N = 6.15%.

*7-Methoxy-3-methyl-2-[2-(3,4,5-trimethoxyphenyl)cyclopropanecarbonyl] quinoxaline 1,4-di-*N*-oxide* (**4c**). Yield 13%. mp 174–175 °C. ^1^H-NMR (DMSO-*d_6_*) δ ppm: 1.84 (m, 2H, H_c_+H_c'_); 2.37 (s, 3H, CH_3_); 2.79 (m, 1H, H_a_); 2.90 (m, 1H, H_b_); 3.61 (s, 3H, *p*-OCH_3_); 3.77 (s, 6H, *m*-OCH_3_); 3.98 (s, 3H, 7-OCH_3_); 6.54 (s, 2H, H_2'_+H_6'_); 7.58, 7.61 (dd, 1H, H_6_, *J*_6–8_ = 2.8 Hz, *J*_6–5_ = 9.5 Hz); 7.74 (d, 1H, H_8_, *J*_8–6_ = 2.7 Hz); 8.40 (d, 1H, H_5_, *J*_5–6_ = 9.5 Hz). IR (KBr) ν cm^−1^: 3005 (w, ν_arC-H_); 2945 (w, ν_C-H_); 1689 (m, ν_C=O_); 1587 (m, ν_arC=C_); 1323 (s, ν_N-O_); 1120 (m, ν_C-O-C_). Anal. Calc. for C_23_H_24_N_2_O_7_: C = 62.71%, H = 5.50%; N = 6.36%; Found: C = 62.55%, H = 5.83%; N = 6.64%.

*3,6,7-Trimethyl-2-[2-(3,4,5-trimethoxyphenyl)cyclopropanecarbonyl] quinoxaline 1,4-di-*N*-oxide* (**4d**). Yield 24%. mp 187–189 °C. ^1^H-NMR (DMSO-*d_6_*) δ ppm: 1.84 (m, 2H, H_c_+H_c'_); 2.37 (s, 3H, CH_3_); 2.47–2.51 (m, 6H, 6,7-CH_3_); 2.76 (m, 1H, H_a_); 2.90 (m, 1H, H_b_); 3.59 (s, 3H, *p*-OCH_3_); 3.75 (s, 6H, *m*-OCH_3_); 6.54 (s, 2H, H_2'_+H_6'_); 8.18 (s, 1H, H_8_); 8.25 (s, 1H, H_5_). IR (KBr) ν cm^−1^: 2932 (w, ν_C-H_); 1683 (m, ν_C=O_); 1589 (m, ν_arC=C_); 1326 (s, ν_N-O_); 1132 (s, ν_C-O-C_). Anal. Calc. for C_24_H_26_N_2_O_6_: C = 65.73%, H = 5.99%; N = 6.39%; Found: C = 65.41%, H = 5.79%; N = 6.40%.

*3-Methyl-2-[2-(2-naphthyl)cyclopropanecarbonyl] quinoxaline 1,4-di-*N*-oxide* (**5a**). Yield 66%. mp 176–177 °C. ^1^H-NMR (DMSO-*d_6_*) δ ppm: 1.93 (m, 1H, H_c_); 1.99 (m, 1H, H_c'_); 2.40 (s, 3H, CH_3_); 3.01 (m, 2H, H_a_+H_b_); 7.38 (d, 1H, H_3’_); 7.47 (m, 2H, H_6'_+H_7'_); 7.80 (s, 1H, H_1'_); 7.85 (m, 3H, H_4'_+H_5'_+H_8'_); 7.96 (m, 2H, H_6_+H_7_); 8.42 (d, 1H, H_8_, *J*_8–7_ = 8.2 Hz); 8.48 (d, 1H, H_5_, *J*_5–6_ = 8.5 Hz). IR (KBr) ν cm^−1^: 3091 (w, ν_arC-H_); 2959 (w, ν_C-H_); 1689 (s, ν_C=O_); 1597 (w, ν_arC=C_); 1328 (s, ν_N-O_). Anal. Calc. for C_23_H_18_N_2_O_3_: C = 74.57%, H = 4.91%; N = 7.56%; Found: C = 74.57%, H = 4.90%; N = 7.57%.

*3-Methyl-2-[2-(3,4,5-trimethoxyphenyl)cyclopropanecarbonyl] quinoxaline* (**6a**). Yield 41%. mp 155–156 °C. ^1^H-NMR (DMSO-*d_6_*) δ ppm: 1.77 (s, 1H, H_c_); 1.84 (s, 1H, H_c'_); 2.69 (s, 1H, H_a_); 2.86 (s, 3H, CH_3_); 3.57 (m, 1H, H_b_); 3.61 (s, 3H, *p*-OCH_3_); 3.77 (s, 6H, *m*-OCH_3_); 6.59 (s, 2H, H_2'_+H_6'_); 7.88 (m, 1H, H_6_); 7.96 (m, 1H, H_7_); 8.08 (m, 1H, H_8_); 8.18 (m, 1H, H_5_). IR (KBr) ν cm^−1^: 2961 and 2935 (w, ν_C-H_); 1679 (s, ν_C=O_); 1589 (m, ν_arC=C_); 1132 (s, ν_C-O-C_). Anal. Calc. for C_22_H_22_N_2_O_4_: C = 69.81%, H = 5.87%; N = 7.40%; Found: C = 69.86%, H = 5.83%; N = 7.40%.

*7-Methoxy-3-methyl-2-[2-(3,4,5-trimethoxyphenyl)cyclopropanecarbonyl] quinoxaline* (**6c**). Yield 29%. mp 163–164 °C. ^1^H-NMR (DMSO-*d_6_*) δ ppm: 1.76 (m, 1H, H_c_); 1.84 (m, 1H, H_c'_); 2.69 (m, 1H, H_a_); 2.83 (s, 3H, CH_3_); 3.58 (m, 1H, H_b_); 3.63 (s, 3H, *p*-OCH_3_); 3.80 (s, 6H, *m*-OCH_3_); 3.96 (s, 3H, 7-OCH_3_); 6.60 (s, 2H, H_2'_+H_6'_); 7.52 (d, 1H, H_8_, *J*_8–6_ = 2.8 Hz); 7.58, 7.60 (dd, 1H, H_6_, *J*_6–8_ = 2.8 Hz, *J*_6–5_ = 9.2 Hz); 7.98 (d, 1H, H_5_, *J*_5–6_ = 9.2 Hz). IR (KBr) ν cm^−1^: 2936 (w, ν_C-H_); 1680 (s, ν_C=O_); 1587 (m, ν_arC=C_); 1130 (s, ν_C-O-C_). Anal. Calc. for C_23_H_24_N_2_O_5_: C = 67.62%, H = 5.93%; N = 6.86%; Found: C = 67.23%, H = 6.13%; N = 6.74%.

### 3.3. *In Vitro* Antiplasmodial Drug Assay

Chloroquine-resistant FCR-3 strain of *P. falciparum* was cultivated at 37 °C in 5% CO_2_, 5% O_2_ in a balanced N_2_ atmosphere environment on RPMI 1640 medium supplemented with gentamicin 0.1 mg/mL and 10% heat-inactivated A^+^ human serum, as previously described [59]. The drugs, dissolved in dimethyl sulfoxide, were added at final concentrations ranging from 250 to 0.1 μM. The final DMSO concentration was never greater than 0.1%. *In vitro* antimalarial activity was measured using the [3H]-hypoxanthine (MP Biomedicals, Santa Ana, CA, USA) incorporation assay [60]. Briefly, 250 μL of total culture medium with the diluted drug and the suspension of human red blood cells in medium (A^+^ group, 5% hematocrit) with 1% parasitemia were placed into the wells of 96-well microtiter plates. On the third day of the test, radioactivity was assessed. All experiments were performed in triplicate. Results were expressed as the concentration resulting in 50% inhibition (IC_50_), which was calculated by a nonlinear regression logistic dose response model. The mean IC_50_ values and standard deviation for each compound was calculated.

## 4. Conclusions

Eighteen quinoxaline and quinoxaline 1,4-di-*N*-oxide derivatives have been synthesized with the aim of studying their antimalarial activity. The SAR study suggested that the chalcone and inverted chalcone moieties can act as useful linkers in the search for antimalarial ligands. Moreover, the similar activities of series 1, 3 and 7 allow us to affirm that the enone moiety plays an important role in the antimalarial activity of these compounds, but not its orientation. Compounds **1a** and **2a** showed the best antiplasmodial activity. In general, the addition of cyclopropyl moiety dramatically reduces the biological activity in each case. We are not able to unequivocally confirm that *N*-oxides are essential for the antiplasmodial activity. These results show that further structural modifications may provide better antimalarial compounds.
